# A Clinical Study of Decellularized Tissue Engineering Hyaline Cartilage Graft (dLhCG) for Articular Cartilage Repair

**DOI:** 10.1002/advs.202523106

**Published:** 2026-10-13

**Authors:** Zhen Zhang, Gong Cheng, Wenyong Fei, Qiuzhen Liang, Liang Zhang, Kegui Liu, Jihang Dai, Bo Cong, Xu Hu, Zhonglian Wu, Jialu Zhang, Jinlan Cui, Hang Yao, Dong‐An Wang, Jingcheng Wang, Tao Sun, Jiang Zheng

**Affiliations:** ^1^ Department of Biomedical Engineering City University of Hong Kong Kowloon Tong Hong Kong SAR People's Republic of China; ^2^ Department of Osteoarthropathy Yantaishan Hospital Yantaishan Hospital Affiliated to Binzhou Medical University Yantai Yantai Shandong People's Republic of China; ^3^ Department of Sports Medicine Northern Jiangsu People's Hospital Yangzhou Jiangsu People's Republic of China; ^4^ Sports Medicine Center Honghui Hospital Xi'an Jiaotong University Xi'an Shanxi People's Republic of China; ^5^ Center of Basic and Clinical Research in Sports Medicine Yangzhou University Yangzhou Jiangsu People's Republic of China; ^6^ Yantai Dinghao Biotechnology Co., Ltd. Yantai Shandong People's Republic of China; ^7^ Department of Biomedical Engineering The Chinese University of Hong Kong Sha Tin Hong Kong SAR People's Republic of China

**Keywords:** articular cartilage repair, clinical study, dLhCG, type II collagen

## Abstract

Current clinical approaches for knee cartilage repair are often limited by hypertrophy and fibrosis in the regenerated tissues. A decellularized tissue‐engineered hyaline cartilage graft (dLhCG), composed of a type II collagen‐based extracellular matrix, has been developed to overcome this limitation. Extensive preclinical studies have demonstrated the ability of dLhCG to achieve hyaline cartilage repair and maintain a stable hyaline phenotype. In this prospective, first‐in‐human, exploratory clinical study (ChiCTR2400094194, ChiCTR2500100526, and ChiCTR2500105847), dLhCG was implanted to treat full‐thickness knee articular cartilage defects (Outerbridge grade III/IV) in 17 patients. Stable graft integration with the surrounding native tissue was observed up to 18 months postoperatively. Significant improvements were noted in clinical outcome scores and MOCART scores. MRI T2 mapping evaluated as the ratio of repair‐tissue echo time to adjacent healthy cartilage, yielded values approaching 1, suggesting hyaline‐like biochemical and structural characteristics. No serious clinical adverse events were reported; asymptomatic imaging abnormalities, including bone marrow edema, were noted in some cases. These findings provide preliminary evidence of the feasibility, short‐term safety, and encouraging outcomes of dLhCG for hyaline‐like cartilage repair, warranting further investigation in controlled clinical trials.

## Graphical abstract

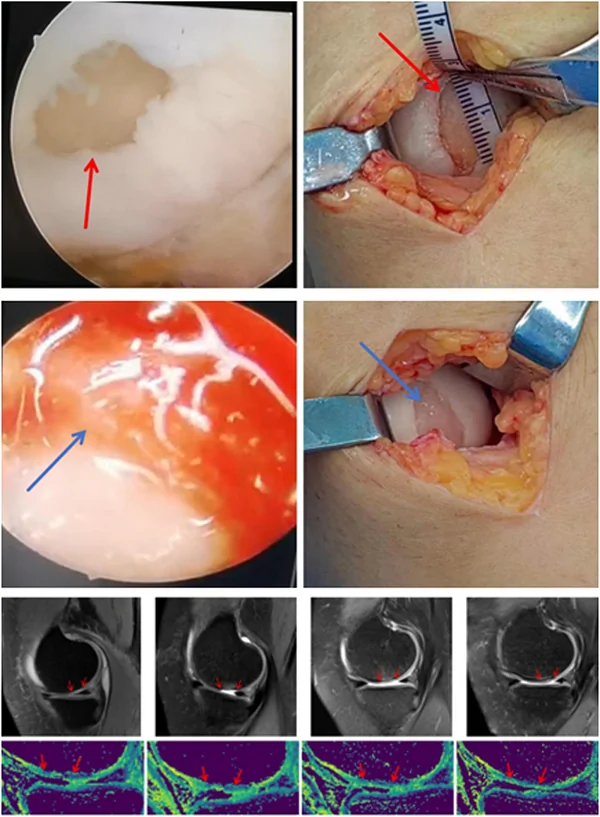

## 1 Introduction

Articular cartilage comprises chondrocytes and an extracellular matrix (ECM), with Type II collagen accounting for 95% of the total collagen content and 60% of the cartilage's dry weight in mature articular cartilage [1]. The ECM is also rich in proteoglycans, glycosaminoglycans (GAGs), and water, collectively maintaining the specific articular cartilage functions, such as load‐bearing, pressure dispersion, and friction reduction [2]. Cartilage injury represents a common pathological hallmark of osteoarthritis (OA). This widespread degenerative condition stands as a primary cause of disability, impacting over 303 million individuals globally [3] and projected to increase by 50% in the next few years [4]. The progression of OA and cartilage defects mutually influences each other, with cartilage injuries leading to joint function deterioration, abnormal stress distribution, and the onset of OA. Conversely, OA results in the degradation of the articular cartilage matrix, further worsening cartilage injuries. Hence, the primary focus in treating cartilage injuries is addressing these defects. Articular cartilage, devoid of blood vessels, nerves, or lymph nodes, exhibits limited self‐repair capabilities following damage, often leading to knee pain and functional impairment [1, 5]. Presently, common clinical treatment modalities for cartilage damage comprise microfracture (MF), autologous osteochondral transplantation (OATS), allogeneic osteochondral transplantation (OCA), and autologous chondrocyte transplantation (ACI). However, these techniques are hindered by subchondral bone injury (MF), fibrous repair, donor scarcity, and inadequate tissue integration [2]. In response to these challenges, biomimetic scaffolds and tissue engineering strategies have been extensively investigated for both bone and cartilage regeneration, employing advanced materials and bioactive factors to improve repair outcomes [6, 7].

Collagen, a vital constituent of the ECM in articular cartilage, is a promising scaffold material for cartilage repair [1, 8]. Type II collagen, a key element of articular cartilage, demonstrates superior potential for hyaline cartilage regeneration compared to type I and type III collagen [9]. However, type II collagen has a complex and costly separation and purification process. In contrast, type I collagen has gained popularity as a scaffold material for articular cartilage repair [5, 8, 9]. However, numerous clinical studies have revealed that type I and type III collagen scaffolds often lead to cartilage fibrosis and hypertrophy within the repair site, thwarting their practical clinical application. Our team aims to design and produce an implantable material for achieving hyaline‐like cartilage repair and regeneration using tissue engineering approaches. Since the ECM components of hyaline cartilage are primarily composed of type II collagen secreted by chondrocytes, specific steps must be followed in this process. In our previous works [10, 11], chondrocytes were cultured on microcarriers with controlled size and porosity, which promoted cell adhesion and the characteristic “edge flourish” growth pattern, facilitating extensive type II collagen‐rich ECM deposition. This enabled the establishment of a suitable 3D culture environment in vitro that supports the proliferation of chondrocytes and induces their substantial secretion of hyaline cartilage‐specific matrix. To ensure the low immunogenicity of the implant material, the obtained hyaline cartilage grafts from in vitro preparation need to undergo decellularization processing. Moreover, to prevent the transmission of animal‐derived pathogens, all production steps are performed under strictly controlled conditions that comply with relevant medical device standards. In preceding work by Su et al. [12], living hyaline cartilage grafts (LhCG) were synthesized without non‐cartilage components, yielding a decellularized tissue engineering hyaline cartilage graft (dLhCG) primarily comprised of type II collagen. Following ongoing enhancements to the production process, dLhCG has successfully transitioned into industrial‐scale manufacturing.

This study represents a first‐in‐human, prospective, exploratory investigation of a type II collagen‐based scaffold for the repair of full‐thickness knee cartilage defects. The primary objective was to evaluate the preliminary feasibility, short‐term safety, and imaging‐based outcomes of dLhCG, as assessed by clinical scores and quantitative magnetic resonance imaging (MRI).

## 2 Results

### 2.1 Material Characteristics

The construction of LhCG utilizes tissue engineering techniques to establish a 3D growth and proliferation platform for chondrocytes in vitro, harnessing the secretory function of these cells to create a hyaline cartilage graft devoid of non‐cartilaginous components. The inspiration behind LhCG development came from the “Edge Flourish” phenomenon observed when cultivating chondrocytes in hydrogels, where chondrocytes at the edge of the hydrogel exhibit abundant new tissue growth and a distinct hyaline cartilage phenotype compared to the interior of the hydrogel. Based on this “Edge Flourish” phenomenon, a scaffold technology (Figure 1A) employing microcavity hydrogels and a novel phase transfer cell culture strategy was employed to prepare LhCG [12]. In essence, the dissolution of gelatin microspheres generates microcavities that provide numerous interfaces for chondrocytes within the hydrogel, facilitating “Edge Flourish” growth.

**FIGURE 1:**
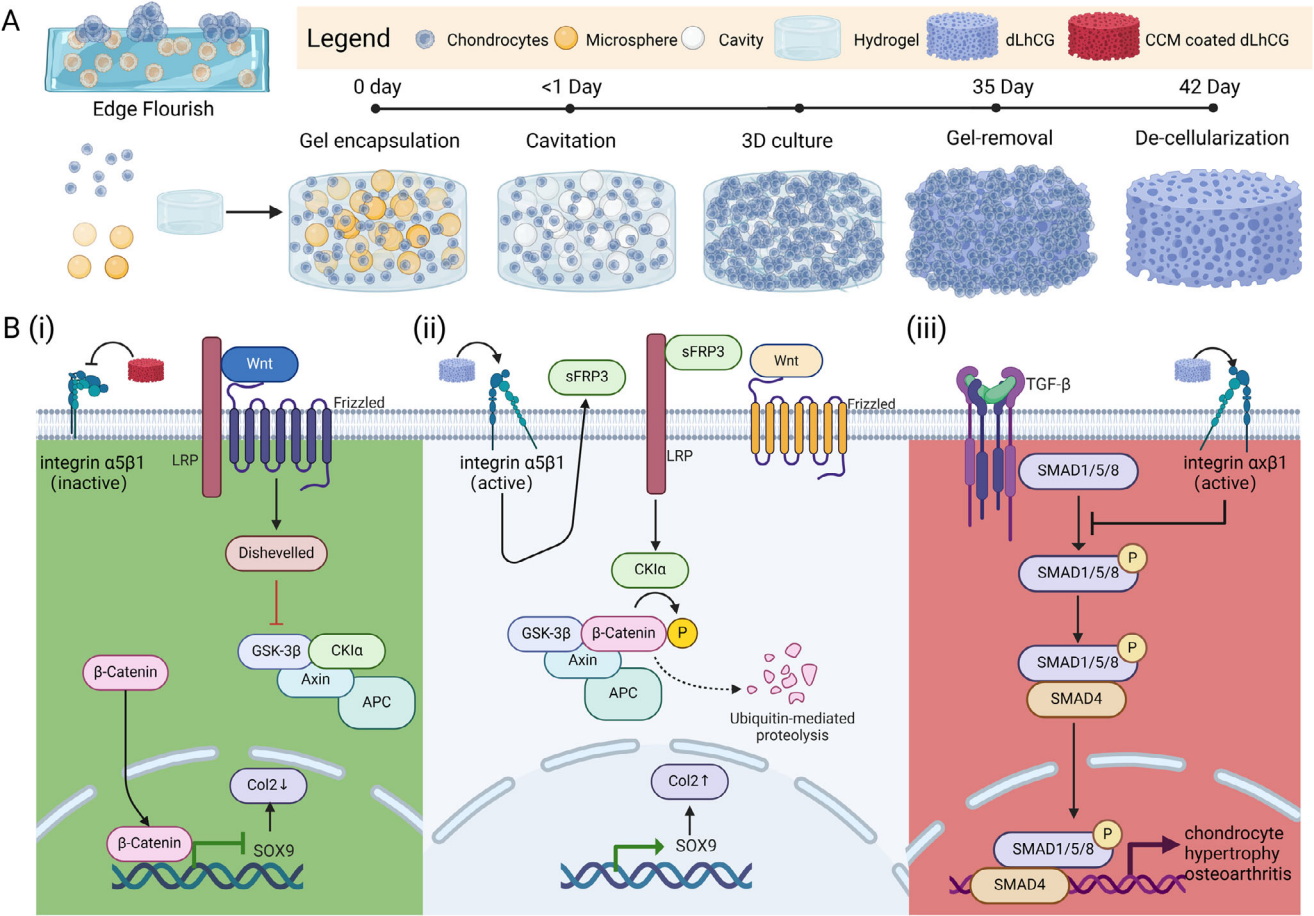
The dLhCG preparation process and the proposed mechanism of hyaline cartilage repair facilitated by dLhCG. (A) In the dLhCG preparation process, the design of dLhCG draws inspiration from the Edge Flourish concept, where microcavities provide numerous interfaces for chondrocytes to exhibit robust growth and maintain a hyaline cartilage phenotype. (B) Mechanism of cartilage repair: (i) On dLhCG coated with chondrocyte membrane (CCM), the active site is shielded, preventing the activation of integrin α5β1. This inhibits the ubiquitination and hydrolysis of β‐Catenin mediated by LRP and Wnt signaling, subsequently blocking the transcription of SOX9 and reducing Col2 production. (ii) Conversely, the active site on unmodified dLhCG can activate integrin α5β1, prompting cells to secrete sFRP3. sFRP3 binds to LRP, initiating the ubiquitination and hydrolysis of β‐Catenin, enabling normal transcription of SOX9 for Col2 production. (iii) In the osteoarthritis (OA) environment, the active site on dLhCG can activate integrin αxβ1, inhibiting cartilage hypertrophy and osteogenesis induced by the TGF‐β‐SMAD1/5/8 signaling pathway.

From LhCG to dLhCG, the decellularization process resulted in the nearly complete removal of relatively highly hydrophilic, relatively low‐molecular‐weight, non‐crosslinked components. Specifically, nucleic acids (double‐stranded assay) decreased from 91.56 ± 22.56 µg per mg total dry weight in LhCG to only 20.59 ± 4.22 ng per mg total dry weight in dLhCG (provided by EpinTek (Guiyang) Quality Technology Co., Ltd. as the minimally measured value), and no intact nuclei were observed; meanwhile, GAG decreased from 18.45 ± 3.03 µg per mg total dry weight in LhCG to an undetectable level in dLhCG (provided by Standard Sci‐Tech Medical Technology (Qingdao) Co., Ltd. as the minimally measured value). Consequently and in contrast, collagen, as the naturally crosslinked and assembled supramolecular component of high‐molecular‐weight biomacromolecules, increased from 528.96 ± 43.92 µg per mg total dry weight in LhCG to nearly 100% total dry weight in dLhCG (provided by Yantai Dinghao Biotechnology Co., Ltd.). The scaffold exhibited an interconnected porosity of 75.21  ±  4.27%. The surface pore size is 163.6  ±  35.44 µm, and the cross‐sectional pore size is 122.7 ± 17.79 µm (n = 3). Immunohistochemistry confirmed the preservation of type II collagen, with negligible type I collagen. In vitro characterization further demonstrated that the dLhCG scaffold possessed a compressive modulus of 10.93  ±  3.55 kPa (at 20% strain; n = 4). Dynamic mechanical analysis at 1 Hz gave storage moduli (G′) of 1243  ±  437.9 Pa, and loss moduli (G″) of 256.4 ± 82.73 Pa (Figure S1).

The test report for dLhCG is as follows: The appearance is white or slightly yellowish, in the form of membranous sheets with an average diameter of 27.38 mm and an average thickness of 0.69 mm. The pH value is 6.3, and the hydroxyproline content accounts for approximately 13% of the sample's dry weight. The sample is sterile, with bacterial endotoxin levels less than 20 EU per unit, heavy metal content below thirty parts per million, and the decellularization process meets the established technology standards for medical devices. All the above indicators meet the requirements for medical device technical specifications.

In a preclinical large‐animal study, Peck et al. [13] validated using porcine autologous mature chondrocytes‐generated LhCG in a mature pig cartilage defect model. After 6 months of implantation, the LhCG demonstrated excellent macroscopic repair and integration with the surrounding cartilage, characterized by maintained cartilage thickness and restoration of the joint surface integrity. Notably, the repair tissue produced by LhCG was biologically equivalent to native hyaline cartilage. Nie et al. [14, 15] found that porcine‐derived dLhCG transplantation could achieve cartilage defect regeneration with a correct hyaline cartilage phenotype in pig and rabbit models. Furthermore, this repair was significantly superior to that achieved with ACI. Research by Zhou et al. [16] indicated that adding the chondrocyte membrane coating on dLhCG scaffolds antagonized the integrin α5 receptor on the chondrocytes, preventing its binding to corresponding ligand sites on the type II collagen in the dLhCG scaffold, which would otherwise promote Wnt/β‐catenin signaling, leading to the loss of the hyaline cartilage phenotype (Figure 1B‐i). This finding reveals that dLhCG can maintain the hyaline cartilage phenotype by inhibiting the Wnt/β‐catenin signaling pathway (Figure 1B‐ii). In a study by Hu et al. [17], critical and overcritical‐size osteochondral defect models were established in osteoarthritic rats. Transplantation of porcine‐derived dLhCG achieved hyaline cartilage regeneration after 90 and 150 days, indicating that dLhCG may regulate cartilage repair in an osteoarthritic environment by inhibiting the TGF‐β‐Smad1/5/8 signaling pathway (Figure 1B‐iii). Zhang et al. [18] achieved injectable in situ cartilage regeneration in a rat cartilage defect model by micronizing porcine‐derived dLhCG and combining it with bone marrow and thrombin. This provides a reference for minimally invasive treatment, and the injected material was shown to down‐regulate the T cell receptor signaling pathway and IL‐17 signaling pathway in the treated area, thereby altering the immune microenvironment to facilitate hyaline‐like cartilage repair and regeneration. Compared to Type I and Type I/III collagen scaffolds, LhCG/dLhCG, primarily composed of Type II collagen, offers advantages including a closer resemblance to natural hyaline cartilage ECM, maintenance of chondrocytes spheroid morphology, and the presence of bioactive sites for acquiring and sustaining the transparent chondrocytes phenotype.

### 2.2 Clinical Efficacy

#### 2.2.1 Surgical Procedure

The dLhCG scaffold was implanted either by arthroscopic or open surgery. In cases where a defect was present in the femoral condyle, a stable and intact cartilage border was meticulously established around the defect using a sharp metal punch. The lesion was carefully debrided down to the subchondral plate, ensuring the subchondral bone remained unharmed. Subsequently, the dLhCG scaffold was customized to match the size and shape of the lesion. Fibrin glue was applied to the bottom of the cartilage defect before the implant was transferred to the lesion. The specific surgical procedure is shown in Figure 2.

**FIGURE 2:**
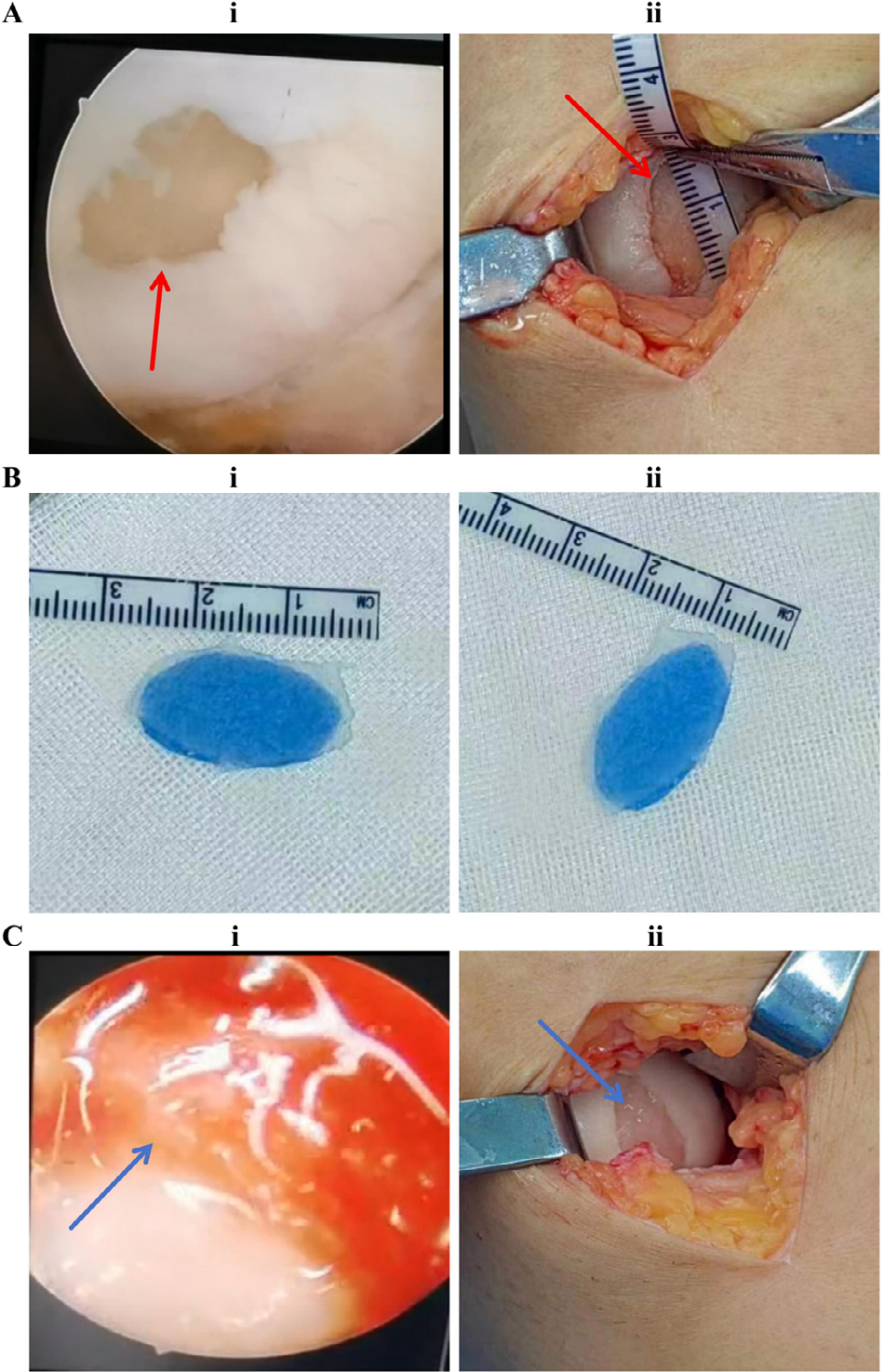
Intraoperative images of dLhCG implantation. (A) Debridement and trimming of cartilage defects (Red Arrow) by arthroscopic surgery (i) or open surgery (ii). (B) Cut appropriate dLhCG according to the shape and size of the defect. (C) Implantation of dLhCG (Blue Arrow) by arthroscopic surgery (i) or open surgery (ii).

#### 2.2.2 Demographic and Baseline Characteristics

From October 2022 to November 2025, seventeen patients were enrolled and analyzed in this study. Seventeen patients underwent follow‐up at 6 and 12‐month post‐surgery; six patients completed a follow‐up period of ≥18 months. The baseline characteristics of the cohort are summarized in Table 1. Preoperatively, the average clinical scores of the 17 patients were as follows: the International Knee Documentation Committee (IKDC) score was 52.64 ± 13.76; the visual analog scale (VAS) pain score was 4.35 ± 2.29, and the Lysholm and Tegner scores (available for 8 patients) were 62.88 ± 19.75 and 3.50 ± 1.69, respectively. The preoperative ratio of cartilage thickness between the regeneration area and the native area (RA/NA) was 0.759 ± 0.20.

**TABLE 1:** Baseline demographic and clinical characteristics of the study cohort.

Characteristic	Mean ±SD
Gender (Male/Female)	7/10
BMI	26.20 ± 3.27
Age	50.06 ± 14.86
Course of Disease (Year)	0.91 ± 1.65
Defect size (cm^2^)	1.22 ± 1.12
Defect location (Left/Right)	7/10
Outerbridge (III/IV)	5/12
Surgical duration (Minutes)	73.77 ± 27.73
Methods of surgery (Arthroscopic/Open)	12/5
Disease diagnosis	
(1) Cartilage defect only	6
(2) With tissue injury	8
(3) With osteoarthritis	3

#### 2.2.3 Clinical and MOCART Scores

The clinical scores and cartilage thickness of the 17 patients were assessed at multiple time points: preoperatively, and 3, 6, 12, and ≥18 months postoperatively (Figure 3 and Table S1). The improvements at each postoperative time point are shown in Table 2 and Table S2. Among the six patients who completed follow‐up at ≥18 months, all attain a VAS score of 0, reflecting complete pain resolution. Concurrently, all functional and structural outcome measures demonstrate continuous improvement over time. IKDC scores show a steady upward trend, reaching 78.35 ± 14.42, while Tegner scores consistently improve to 5.83 ± 2.23 and Lysholm scores improve to 88.50 ± 18.28, indicating sustained recovery of knee function and mobility. Similarly, the Magnetic Resonance Observation of Cartilage Repair Tissue (MOCART) score exhibits progressive improvement, reaching 80.56 ± 7.35. The cartilage thickness ratio (RA/NA) reaches 0.996 ± 0.17. The consistent positive trajectory across all scoring metrics confirms robust and progressive repair following the intervention (Table S1).

**FIGURE 3:**
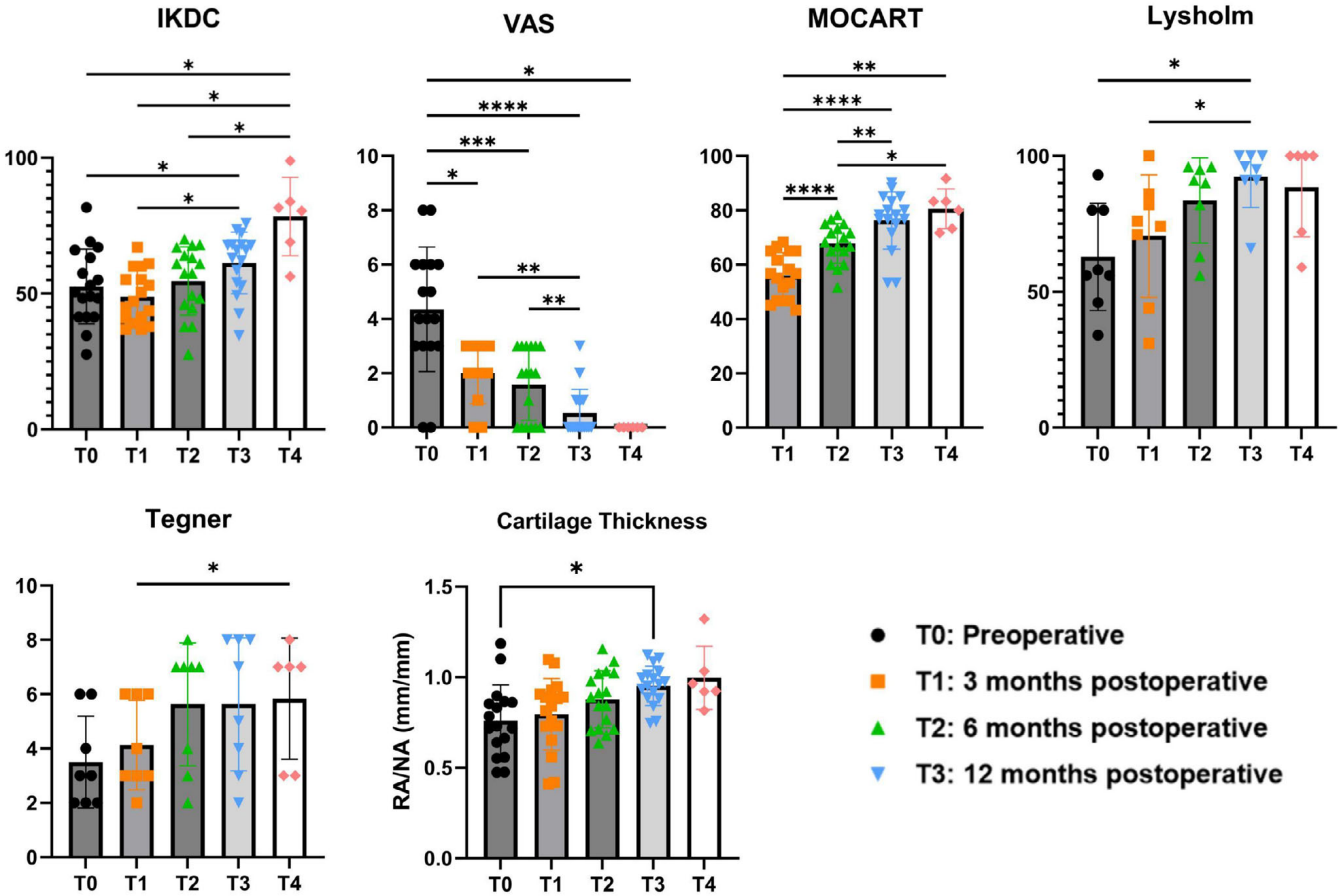
Comparison of preoperative and postoperative IKDC, MOCART, VAS, Lysholm, and Tegner scores, and cartilage thickness. (Mixed‐effects model, ^*^ *P* < 0.05, ^**^ *P* < 0.01, ^***^ *P* < 0.001, and ^****^ *P* < 0.0001).

**TABLE 2:** Comparisons of clinical and MRI outcomes across postoperative time points.

Characteristic	3 months vs Preoperative		6 months vs Preoperative		12 months vs Preoperative	
	Mean (95% Cl)	Adjusted P Value	Mean (95% Cl)	Adjusted P Value	Mean (95% Cl)	Adjusted P Value
**VAS**	−2.35 (−0.22 to −4.49)	0.027	−2.77 (−1.16 to −4.37)	0.0006	−3.82 (−2.18 to −5.47)	<0.0001
**IKDC**	−3.78 (6.78 to −14.33)	0.806	2.00 (11.93 to −7.94)	0.970	8.68 (16.66 to 0.70)	0.030
**Tegner**	0.63 (1.78 to －0.53)	0.383	2.13 (4.41 to −0.16)	0.069	2.13 (4.60 to −0.35)	0.095
**Lysholm**	7.63 (42.42 to −27.16)	0.927	20.75 (46.56 to −5.06)	0.121	29.50 (58.15 to 0.85)	0.044

Characteristic	6 months vs 3 months		12 months vs 6 months		12 months vs 3 months	
	Mean (95% Cl)	Adjusted P Value	Mean (95% Cl)	Adjusted P Value	Mean (95% Cl)	Adjusted P Value
**VAS**	−0.41 (−0.70 to 1.53)	0.789	−1.06 (−0.25 to −1.87)	0.008	−1.47 (−0.52 to −2.42)	0.002
**IKDC**	5.77 (15.56 to −4.01)	0.402	6.68 (16.28 to −2.92)	0.255	12.46 (22.47 to 2.44)	0.012
**Tegner**	1.50 (3.29 to −0.29)	0.103	0.00 (1.66 to −1.66)	>0.9999	1.50 (3.29 to −0.29)	0.103
**Lysholm**	13.13 (35.69 to −9.44)	0.322	8.75 (23.01 to −5.51)	0.282	21.88 (41.15 to 2.60)	0.028
**MOCART**	11.86 (16.38 to 7.35)	<0.0001	8.43 (13.68 to 3.12)	0.0015	20.29 (26.48 to 14.10)	<0.0001

Compared with preoperative scores, the VAS score decreases significantly by 2.35 points (95% CI, 0.22 to 4.49; *P* = 0.027) at 3 months postoperatively. At 6 months, the VAS score decreases by 2.77 points (95% CI, 1.16 to 4.37; *P* = 0.0006). No significant changes are observed in IKDC, Tegner, or Lysholm scores at 3‐ or 6‐months postoperatively. By 12 months, further improvements are evident: the VAS score decreases significantly by −3.82 (95% CI, −2.18 to −5.47; *P* < 0.0001), the Tegner score improves by 2.13 (95% CI, −0.35 to 4.60; *P* = 0.095); the Lysholm score increases significantly by 29.50 (95% CI, 0.85 to 58.15; *P* = 0.044); and the IKDC score improves by 8.68 (95% CI, 0.70 to 16.66; *P* = 0.03). The MOCART score increases from 55.98 ± 8.36 at 3 months to 67.84 ± 7.28 at 6 months, and further to 76.27 ± 10.53 at 12 months. Analysis of inter‐time point changes (Table 2) reveals that the reduction in pain occurs significantly between 6 and 12 months after surgery, with a VAS decrease of 1.06 points (95% CI, 0.25 to 1.87; *P* = 0.008). For the MOCART score, a significant improvement is observed between 3 and 6 months postoperatively, with an increase of 11.86 (95% CI, 7.35 to 16.38; *P* < 0.0001). The IKDC, Tegner, and Lysholm scores, as well as the cartilage thickness ratio, demonstrate progressive improvement over time.

The individual longitudinal trajectories of clinical, MRI score, and cartilage thickness outcomes for all 17 patients across the preoperative and postoperative follow‐up period are presented in Figure 4. A consistent trend of improvement is observed across all six key metrics. The IKDC and Lysholm scores demonstrate a pronounced progressive increase from baseline, indicating substantial gains in knee‐specific function and stability. Concurrently, the VAS score exhibits a marked progressive decline, reflecting an effective reduction in pain. The Tegner activity scale shows a more gradual but positive upward trajectory, signifying a steady return to higher physical activity levels. Structural assessment via the MOCART score and the cartilage thickness ratio (RA/NA) reveals progressive improvement, confirming favorable morphological repair and integration of the implanted biomaterial over time. Notwithstanding this overall positive trend, the plots reveal a degree of heterogeneity in individual patient responses. This heterogeneity is further investigated in the following sections using cluster analysis, correlation analysis, and linear regression.

**FIGURE 4:**
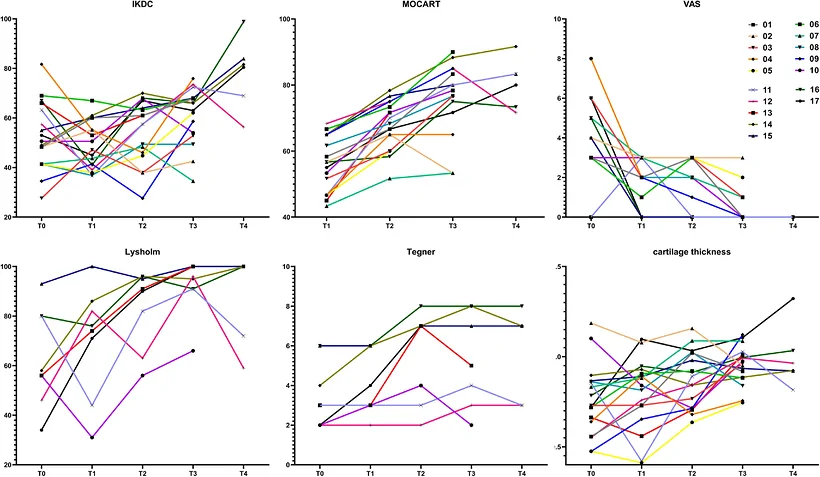
The individual longitudinal trajectories of IKDC, MOCART, VAS, Lysholm, Tegner scores, and Cartilage thickness for patients. (T0: Preoperative. T1: 3 months postoperative. T2: 6 months postoperative. T3: 12 months postoperative. T4: ≥18 months postoperative.).

#### 2.2.4 Evaluation of Regenerated Cartilage Tissue

Compared with preoperative MRI, a progressive enhancement in the repair efficacy of dLhCG at the defect site is observed over time (Figure 5A,B). At 12 months postoperatively, MRI demonstrates complete filling of the defect site by dLhCG, with successful integration into the surrounding tissue. The regenerated tissue exhibits a smooth surface; its internal structure and signal intensity resemble normal cartilage. The subchondral bone plate appears continuous and structurally sound. Although some residual subchondral edema persists, it is notably diminished compared with the preoperative state. MRI images for all cases are shown in Figure S2.

**FIGURE 5:**
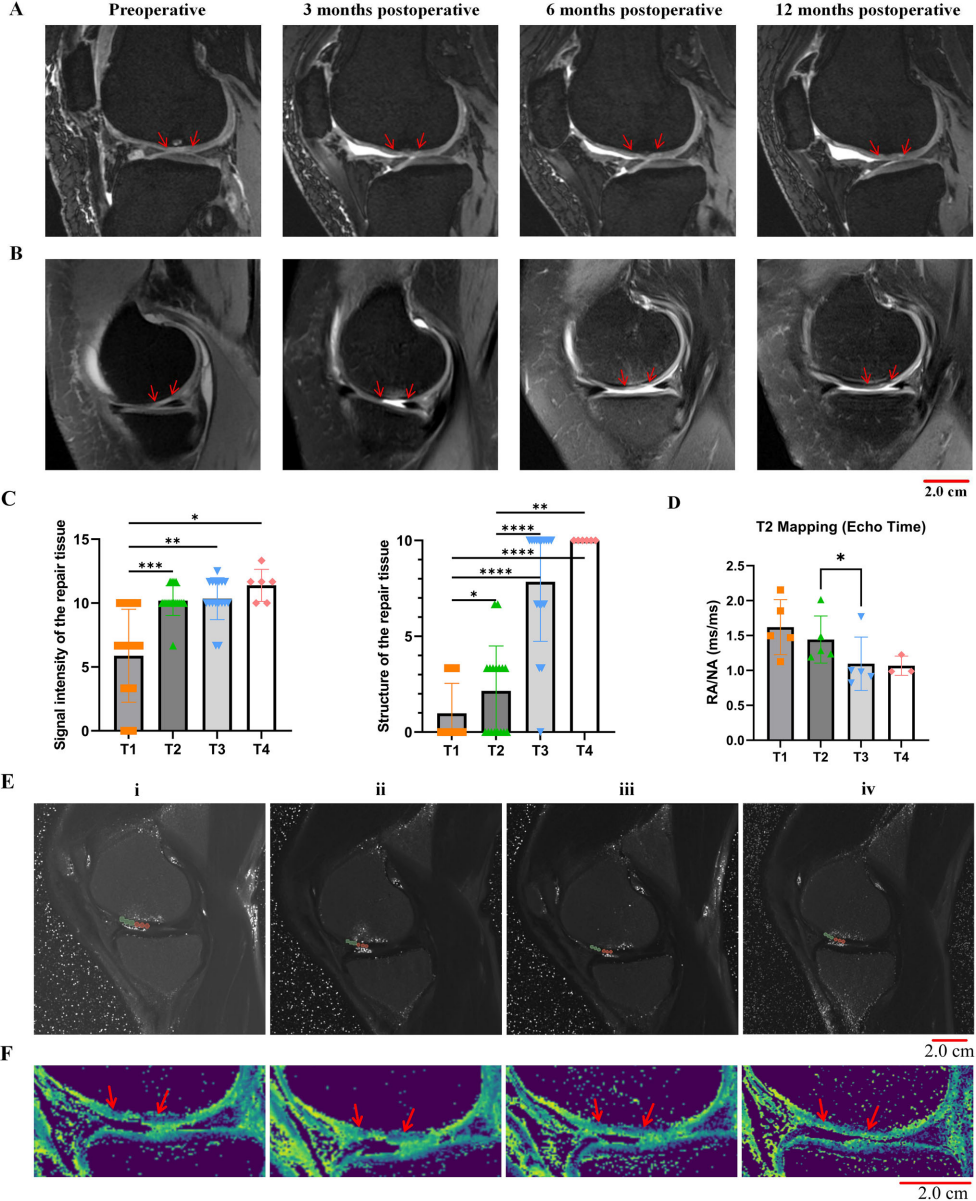
MRI images of a typical cartilage defect on the femoral condyle before and after dLhCG transplantation. Open surgery (A) or arthroscopic surgery (B). The red arrow indicates the cartilage defect region or the dLhCG implantation site. (C) The scores for signal intensity and structure of the repair tissue on MRI (T0, Preoperative; T1, 3 months postoperative; T2, 6 months postoperative; T3, 12 months postoperative; T4, ≥18 months postoperative). (D) The Echo time analysis based on MRI T2 Mapping (*n* = 5 at T1 to T3, *n* = 3 at T4). (E) The MRI T2 Mapping of patient 15 (The green area is native cartilage, and the red area is regenerated cartilage), (i) 3 months postoperative, (ii) 6 months postoperative, (iii) 12 months postoperative, (iv) 18 months postoperative. (F) Local magnification pseudocolor image based on T2 mapping, with the area indicated by the red arrow representing the regenerated region. (Mixed‐effects model, ^*^ *P* < 0.05, ^**^ *P* < 0.01, ^***^ *P* < 0.001, and ^****^ *P* < 0.0001. Scale bar = 2.0 cm).

The structural (up to 10) and signal intensity (up to 15) properties of the repair tissue were quantitatively assessed using MRI‐based scoring criteria. A homogeneous structure (score = 10) is defined by the formation of typical cartilage layers throughout the repair tissue or a uniformly appearing tissue. Conversely, an inhomogeneous structure (score = 0) is characterized by disorganized tissue with altered signal intensity, indicating structural heterogeneity. The signal intensity score reflects the biochemical composition of the repaired tissue, particularly its resemblance to the adjacent native hyaline cartilage on T2‐weighted or proton density‐weighted sequences. A higher score indicates a near‐normal, homogeneous signal, suggesting mature cartilage with adequate proteoglycan content and collagen organization, whereas a lower score may indicate fibrocartilage or ongoing remodeling. The MRI scores indicate continuous improvement in structural and signal intensity of the repaired tissue over time, with outcomes converging closely with those of the surrounding native cartilage (Figure 5C). At the 12‐month follow‐up, the structural score reaches 7.84 ± 3.11, and the signal intensity score improves to 10.34 ± 1.64, demonstrating nearly complete morphological and compositional restoration. At the ≥18‐month follow‐up, the structural score reaches 10.00 ± 0.00, and the signal intensity score improves to 11.39 ± 1.26, indicating further morphological and compositional restoration.

T2 mapping has emerged as a valuable non‐invasive imaging technique for the clinical assessment of articular cartilage, enabling distinction among hyaline cartilage, fibrocartilage, and hypertrophic cartilage based on T2 relaxation times, which reflect collagen organization and water content. Hyaline cartilage typically exhibits higher T2 values, generally ranging between 40 and 70 milliseconds, due to its structured collagen matrix and elevated hydration [19, 20, 21, 22], whereas fibrocartilage and hypertrophic cartilage demonstrate lower T2 values, primarily due to their disorganized collagen architecture and reduced water content [22, 23]. This ability to non‐invasively discriminate cartilage phenotypes makes T2 mapping particularly valuable in evaluating repair tissue after surgical intervention. The reliability of T2 mapping in depicting cartilage microstructure has made it instrumental in early diagnosis, longitudinal monitoring, and objective evaluation of repair outcomes [19, 22]. In the context of cartilage repair, T2 mapping allows quantitative tracking of the graft's maturation and its evolution toward a phenotype resembling native hyaline cartilage. The following case illustrates this phenotypic progression, demonstrating how serial T2 mapping can objectively capture the structural integration of regenerated tissue over time (Figure 5E).

Quantitative MRI T2 mapping analysis (Figure 5D), referenced against normal hyaline cartilage around the graft, demonstrates a clear phenotypic evolution of the graft. The echo time ratio between regenerated tissue (RA) and native tissue (NA) is 1.62 ± 0.39 at 3 months, 1.44 ± 0.34 at 6 months, 1.10 ± 0.38 at 12 months, and 1.07 ± 0.14 at ≥18 months. Local magnification pseudocolor images based on T2 mapping indicate the following: (1) Continuity evaluation: at 3 months, a colorless missing area is visible in the center of the repair zone, indicating infiltration of high‐water‐content tissue; by 6 months, the missing area shrinks; at 12 months, the color of the repaired tissue is continuous and intact. (2) Water content evaluation: at 3 months, the visible area is mainly yellow, turning blue‐green by 18 months, indicating a significant decrease in water content and good collagen remodeling. This normalization of quantitative parameters indicates that the regenerated tissue has matured and now exhibits T2 mapping values approaching those of hyaline cartilage, suggesting successful restoration of its biochemical and structural properties.

#### 2.2.5 Exploratory Analysis

Given the small sample size and the limited availability of some clinical outcome measures at certain follow‐up time points, all analyses presented in this section were conducted in a strictly exploratory framework. These analyses were intended to generate hypotheses rather than to draw confirmatory conclusions.

##### 2.2.5.1 Correlation and Linear Regression Analyses

Associations between the independent variables and the dependent variables (all score changes) were assessed using correlation analysis. The correlation analysis indicated that gender, BMI, age, defect size, disease diagnosis, surgical method, and duration of surgery were associated with postoperative scores, and therefore linear regression analyses were performed. The regression results for all scores are presented in Table 3. In the linear regression models, categorical variables were coded as follows and entered as continuous predictors: gender was coded as 1 for male and 2 for female, BMI was coded as 1 for >26 and 2 for ≤26, age was coded as 1 for <45 years old and 2 for ≥45 years old, defect size was coded as 1 for <2 cm^2^ and 2 for ≥2 cm^2^, disease diagnosis was coded as 1 for cartilage defect only, 2 for cartilage defect with tissue injury, and 3 for cartilage defect in OA, surgical method was coded as 1 for arthroscopic surgery and 2 for open surgery, and duration of surgery was coded as 1 for ≤1 h and 2 for >1 h. During the rehabilitation period, the improvement in IKDC from preoperatively to 6 months postoperatively is associated with duration of surgery (R^2^ = 0.26, *P* = 0.022) in this exploratory model. The negative correlation indicates that a longer operative time may be associated with less improvement in IKDC scores. The change in IKDC score from 6 to 12 months is associated with BMI and gender (R^2^ = 0.44, *P* = 0.043). The change in VAS score from 6 to 12 months is associated with surgical method (R^2^ = 0.38, *P* = 0.005). The change in MOCART score from 6 to 12 months is associated with BMI (R^2^ = 0.20, *P* = 0.043), suggesting that higher BMI may be linked to attenuated improvements in both IKDC and MOCART scores. Exploratory analyses of Lysholm and Tegner scores indicate that younger age, shorter operative time, and less complex disease may be associated with better recovery (Table S3).

**TABLE 3:** Exploratory linear regression analyses of factors associated with changes in IKDC, VAS, and MOCART scores.

Item	IKDC				VAS				MOCART			
	Variable	Coefficient	P	R^2^	Variable	Coefficient	P	R^2^	Variable	Coefficient	P	R^2^
T1 vs T0	Surgical duration	−0.488	0.047	0.19	/				/			
T2 vs T0	Surgical duration	−0.552	0.022	0.26	/				/			
T3 vs T2	BMI	−0.577	0.015	0.29	Surgical method	0.647	0.005	0.38	BMI	−0.495	0.043	0.20
	Gender	0.440	0.043	0.44								

##### 2.2.5.2 K‐Means Cluster and Subgroups Analysis

To explore the heterogeneity in treatment response, K‐means clustering was performed using the relative functional improvement in each score at multiple postoperative time points (e.g., 3, 6, and 12 months). As a data‐driven, hypothesis‐generating approach, this classification suggested two patient subgroups with contrasting recovery patterns (Table S4). Comparisons of baseline demographics between these clusters are presented in Table 4. Chi‐square tests suggest possible associations between cluster membership and several baseline variables: surgical duration for the IKDC clusters, gender for the Lysholm clusters, and age for the Tegner clusters.

**TABLE 4:** Associations between selected baseline characteristics and K‐means‐derived response clusters.

Surgical duration	IKDC			Gender	Lysholm			Age	Tegner		
	Cluster 1	Cluster 2	P		Cluster 1	Cluster 2	P		Cluster 1	Cluster 2	P
≤1 h	7	0	0.026	Male	0	3	0.028	<45	3	1	0.028
>1 h	5	5		Female	4	1		≥45	0	4	

Based on these cluster findings, patients were stratified by surgical duration for further exploratory comparisons. Surgical duration ≤1 h was defined as the short surgery group, and >1 h as the long surgery group. Improvements in IKDC scores from preoperatively to each time point were compared (Table 5). In this exploratory subgroup comparison, the short surgery group shows greater improvement from preoperatively to 3 months and to 6 months than the long surgery group (*P* = 0.029 and *P* = 0.022, respectively), which may suggest that a shorter surgical duration facilitates knee functional recovery. For the Lysholm score, female patients demonstrate greater preoperative‐to‐postoperative improvement than male patients, with a statistically significant difference at 12 months (*P* = 0.037). For the Tegner score, patients younger than 45 years show greater improvement from preoperatively than those aged ≥45 years, reaching statistical significance at 6 months (*P* = 0.014).

**TABLE 5:** Exploratory subgroup comparisons according to surgical duration, gender, and age.

**IKDC**	**3 months vs Preoperative**	**P**	**6 months vs Preoperative**	**P**	**12 months vs Preoperative**	**P**
Short surgery (*n* = 7)	4.26 ± 7.02	0.029	10.56 ± 9.04	0.022	11.45 ± 10.50	0.392
Long surgery (n=10)	−9.40 ± 15.51		−3.99 ± 12.90		6.75 ± 11.02	

**Lysholm**	**3 months vs Preoperative**	**P**	**6 months vs Preoperative**	**P**	**12 months vs Preoperative**	**P**
Male (*n* = 3)	−7.33 ± 16.26	0.263	6.00 ± 8.72	0.117	9.33 ± 2.08	0.037
Female (*n* = 5)	16.60 ± 30.38		29.60 ± 20.72		41.60 ± 20.18	

**Tegner**	**3 months vs Preoperative**	**P**	**6 months vs Preoperative**	**P**	**12 months vs Preoperative**	**P**
<45 years old (*n* = 4)	1.00 ± 1.15	0.298	3.50 ± 1.29	0.014	3.50 ± 1.91	0.060
≥45 years old (*n* = 4)	0.25 ± 0.50		0.75 ± 0.96		0.75 ± 0.50	

#### 2.2.6 Safety and Adverse Events

In this trial, no clinical graft‐related adverse events occurred among the 17 patients. All adverse enents were systematically recorded from informed consent through the final follow‐up, and the clinicians assessed causality. In accordance with the preoperatively approved clinical research protocol, the criteria and scope for adverse event reporting were prospectively defined, and the clinicians adjudicated all events.

Graft‐related complications, including infection, effusion, delamination, reoperation, and clinically relevant MRI abnormalities, were prospectively tracked. Blood biomarkers, comprising creatinine, blood urea nitrogen, aspartate aminotransferase, alanine aminotransferase, white blood cell count, C‐reactive protein, red blood cell count, hemoglobin, platelet count (Cr, Urea, AST, ALT, WBC, CRP, RBC, HGB, and PLT) were measured preoperatively and at 3 and 12 months postoperatively. Although blood biomarker levels fluctuated slightly at 3 months postoperatively, no statistically significant differences were observed between preoperative and 12‐month postoperative values (Figure S3). Throughout the study, no joint effusion, swelling, or erythema was observed.

Bone marrow edema was observed on MRI in some patients. Imaging abnormalities fell into two categories: (1) pre‐existing severe bone marrow edema that improved postoperatively but had not completely resolved by the final follow‐up; and (2) new or worsened edema in patients who had minimal or no preoperative edema. These imaging findings were not classified as clinical adverse events for the following reasons: no patient reported related symptoms or discomfort, and the treating clinicians determined that the observed bone marrow edema was not directly associated with the dLhCG graft.

## 3 Discussion

This study prospectively analyzed the clinical and radiological efficacy of dLhCG in treating knee cartilage defects. While type I and type I/III collagen scaffolds have been extensively studied in clinical settings, clinical research on type II collagen scaffolds for cartilage repair remains limited. Currently, BioCartilage, a micronized allogeneic cartilage extracellular matrix, represents one of the few clinically available type II collagen options [24, 25, 26]; however, the donor supply for BioCartilage is severely limited, and tissue integration is often low. dLhCG, which combines tissue engineering and biomanufacturing, overcomes these limitations. This study sought to comprehensively analyze and report the clinical efficacy of the type II collagen scaffold dLhCG from both clinical and radiological perspectives to address the gap in this research field.

Clinical and radiological scores collectively suggest positive outcomes and significant improvements in clinical and radiological scores at different postoperative time points (Figure 3), indicating the efficacy of the surgical interventions. Cluster analysis, correlation analysis, and linear regression highlighted various correlations between clinical scores and different factors during the postoperative recovery period, shedding light on the intricate relationships between these variables in the context of patient outcomes. These exploratory analyses underscored that critical factors influencing postoperative recovery primarily include gender, age, defect size, and duration of surgery. These findings highlight the essential variables that play a significant role in the recovery process following surgery, providing valuable insights for understanding and optimizing patient outcomes. In a meta‐analysis [27] encompassing type I/III collagen scaffolds for cartilage repair, the Lysholm score showed a significant increase of 35 points one‐year post‐surgery compared to preoperative levels. A study [28] involving 23 cases treated with type I/III collagen scaffolds revealed that at one year after the procedure, the IKDC score was 69.3 ± 15.3, the Tegner score was 4.1 ± 1.4, and the VAS score was 1.7 ± 1.3. Similarly, another study [29] involving 44 cases treated with type I/III collagen scaffolds indicated that at one year postoperatively, the IKDC score was 61.7 ± 20.6, and the VAS score was 0.8 ± 1.5. These clinical scores were in line with the outcomes observed at one year after dLhCG repair: IKDC (61.32 ± 11.33), Tegner (5.63 ± 2.45), Lysholm (92.38 ± 11.30), and VAS (0.53 ± 0.87). Of the six patients who completed follow‐up assessments at 18 months or beyond, all achieved a VAS score of 0, indicating complete resolution of pain. Corresponding improvements were observed in functional outcomes, with IKDC scores reaching 78.35 ± 14.42 and Tegner scores improving to 5.83 ± 2.23, consistent with satisfactory knee function and mobility recovery.

These results suggest that in terms of clinical assessments, the cartilage repair efficacy of dLhCG is on par with, or potentially marginally superior to, that of commercially available type I/III collagen scaffolds. With respect to radiological results, a related study [24] of BioCartilage showed that MRI results at one year after surgery, only 20% of the cases had a completely filled defect, and 10% had signal intensity consistent with surrounding tissue. All cases had subchondral bone destruction, and 90% had incomplete subchondral bone plates. In other studies [25, 26], involving the ankle joint, the MOCART score of the BioCartilage repair group was 73.2, which did not differ significantly from 64.1 in the microfracture control group. Numerous clinical investigations [29, 30] of type I collagen scaffolds indicate that using type I collagen may lead to hypertrophy of the repair tissue. Most patients exhibit subchondral bone abnormalities, such as edema, cysts, sclerosis, and hypertrophy, which may be associated with removal of the calcified cartilage layer before implantation [31]. In previous reports, the MOCART score for type I collagen at one‐year post‐surgery was 71.2 ± 12.9 [28] and 12.3 ± 2.6 (maximum score = 19) [29]. In the present study, the MOCART score for knee cartilage defects treated with dLhCG could reach 76.27 ± 10.53 at one‐year post‐surgery, and no hypertrophy was observed. Notably, MOCART scores of six patients increased steadily to 80.56 ± 7.35 at 18 months or beyond, reflecting stable cartilage repair and regeneration following treatment with dLhCG. As shown in Figure 4, Patients 2 and 12 exhibited a decrease in MOCART scores over the course of repair. Specifically, Patient 2 showed a reduction between 6‐ and 12‐months post‐surgery. Corresponding MRI findings (Figure S2) revealed an expansion of subchondral bone marrow edema and abnormal high signal intensity within the cartilage repair zone at 12 months compared to the 6‐month assessment. Similarly, Patient 12 experienced a decline in MOCART score from 12 to 18 months after surgery, with MRI indicating worsened subchondral bone marrow edema and cyst formation. However, the signal intensity of the repaired cartilage remained stable. These findings suggest that the decline in MOCART score may be primarily associated with progressive subchondral bone changes, potentially related to increased physical activity after surgery. The longitudinal T2 mapping results presented herein demonstrate a clear and statistically significant evolution of the repaired cartilage tissue toward a phenotype that closely resembles native hyaline cartilage. The initially elevated T2 values in the graft region at 3 months post‐surgery are consistent with edematous, hyperhydrated, and disorganized tissue, typical of early repair. The subsequent progressive decline in T2 values, culminating in values statistically indistinguishable from those of adjacent native cartilage at 12 and 18 months, strongly suggests successful biochemical maturation and structural restoration. This normalization reflects favorable collagen architecture and water content, indicating hyaline‐like rather than fibrocartilaginous or hypertrophic tissue. These quantitative MRI findings corroborate the favorable functional outcomes observed and underscore the efficacy of the intervention in achieving biologically robust and durable cartilage repair.

The limitation of this study is the sample size of 17 patients, which, while providing significant insights, restricts the generalizability of our findings. Given this limited sample, the present study should be regarded as exploratory in nature. As a prospective case series, it lacks a control group. The study only documented relatively short‐term clinical and radiological data. While none of the cases included experienced graft failure or required additional surgery, further investigation is necessary to ascertain the efficacy of dLhCG in treating knee cartilage lesions. Larger, well‐controlled trials with expanded sample sizes are essential to validate these preliminary findings. Nevertheless, despite these limitations, this study offers valuable reference data and meaningful insights that can inform future research and clinical practice regarding the use of dLhCG for cartilage repair.

## 4 Conclusions

In this small exploratory cohort, the implantation of dLhCG was associated with a favorable safety profile and preliminary evidence of efficacy for repairing severe articular cartilage defects in the human knee. Clinical outcomes and MRI evaluations suggested significant improvement, with T2 mapping indicating that the regenerated tissue achieved stable integration and exhibited structural properties resembling those of native hyaline cartilage. These promising but preliminary findings suggest that dLhCG may mitigate the fibrosis and hypertrophy commonly encountered with current repair techniques, thereby supporting hyaline cartilage regeneration. However, given the limited sample size, these results should be interpreted as hypothesis‐generating, and larger confirmatory studies are warranted to validate these observations.

## 5 Methods

### 5.1 Material Preparation

Healthy porcine chondrocytes were obtained and a hydrogel culture system was prepared using gelatin, sodium alginate, and other necessary reagents. A custom‐made mold was fabricated to secure the quantitative hydrogel culture system, and calcium chloride solution was applied to induce solidification. Once the hydrogel had fully set, the mold was removed and the construct was transferred to culture medium for 3D cultivation. After the designated culture period, substantial secretion of type II collagen was observed within the hydrogel system. Subsequently, the original hydrogel structure was disintegrated with sodium citrate solution. Decellularization was accomplished by employing surfactants, alkaline solutions, physical vibration cleaning, and other procedures, yielding a cartilage repair material primarily composed of type II collagen. To achieve medical‐grade standards, the material was subjected to Cobalt‐60 γ‐irradiation for sterilization and viral inactivation, followed by double‐layer blister packaging. Detailed steps are provided in the supplementary files.

### 5.2 Clinical Study Design

A prospective, multi‐center clinical study was conducted between October 2022 and November 2025 to evaluate the preliminary efficacy and safety of dLhCG implantation for treating articular cartilage lesions of the knee. The inclusion criteria require patients younger than 70 years with traumatic or degenerative, focal, full‐thickness (Outerbridge grade III/IV) articular cartilage defects of the knee with a surface area of <6 cm^2^, confirmed by preoperative MRI. The exclusion criteria are (1) Knee osteoarthritis with significant joint space narrowing (Kellgren‐Lawrence grade ≥III) or varus malalignment >10°; (2) Combined severe subchondral bone and meniscus injury; (3) Autoimmune disease, systemic infection, or any condition affecting the joint structure; (4) Any previous cartilage repair procedures on the index knee within 12 months; (5) Inability to adhere to the follow‐up schedule; (6) Women who were pregnant, lactating, or planning pregnancy within 12 months of enrollment; (7) Known allergy to collagen or porcine‐derived products, or religious/cultural objections to porcine‐derived medical products; (8) Other contraindications as determined by the treating physician. The institutional review boards at all participating centers approved the study protocol, and all patients provided written informed consent prior to enrollment. The trial was registered at the Chinese Clinical Trial Registry (ChiCTR2400094194, ChiCTR2500100526, ChiCTR2500105847).

The surgical procedure involved meticulous debridement of the defect to the level of the subchondral plate, preserving the subchondral bone, and creation of a stable peripheral cartilage border. The dLhCG scaffold was trimmed to match the defect size and fixed in place with a thin layer of fibrin glue applied to the base and edges of the lesion. Perioperative management and postoperative rehabilitation followed standard protocols for patients undergoing conventional cartilage injury surgery, with no additional requirements specific to the dLhCG scaffold.

Data collection encompassed patient demographic information, preoperative medical history, defect characteristics (size, location, and etiology), surgical procedure details, patient‐reported subjective assessments, and clinical examinations conducted by the surgeons. Evaluations were performed using the IKDC score, Lysholm knee function scores, Tegner activity scale, VAS for pain assessment [32], and MRI with MOCART scores [33]. Follow‐up assessments were conducted at 3, 6, 12, and ≥18 months postoperative. The MOCART score was assessed independently by three experienced orthopedic surgeons. The final score used for analysis represents the average of the three evaluations.

Adverse events were systematically monitored and recorded from informed consent through the final follow‐up. An adverse event was defined as any untoward medical occurrence, irrespective of its causal relationship to the study device. Serious adverse events were defined as events resulting in death, persistent or significant disability, inpatient hospitalization, or requiring intervention to prevent permanent impairment. The investigator assessed the causal relationship of each adverse event to the graft, and all device‐related complications, subsequent re‐operations, and clinically significant MRI abnormalities were documented. Subjects were followed until the resolution or stabilization of any adverse event.

### 5.3 Statistical Analysis

Statistical analysis was performed using SPSS (version 25) and GraphPad Prism (version 9). Normality of continuous variables was assessed using the Shapiro–Wilk test and visual inspection of Q–Q plots. Homoscedasticity was evaluated using Levene's test and by plotting residuals against fitted values. Potential outliers were identified via boxplots and studentized residuals; no extreme values necessitated removal or transformation. Demographic and baseline characteristics were reported as means ± standard deviations. Alterations in IKDC, Lysholm, Tegner, VAS, and MOCART scores were collected repeatedly. To account for within‐subject correlation and missing values, a linear mixed‐effects model was fitted with time as a fixed factor and subject as a random effect. When Mauchly's test indicated violations of sphericity, the Geisser–Greenhouse correction was applied. Following a significant main effect of time, Tukey's honestly significant difference test was used for post‐hoc pairwise comparisons among time points. Model assumptions, normality and homoscedasticity of the residuals were verified through Q–Q plots, homoscedasticity plots, and residual plots. K‐Means cluster analysis to explore the heterogeneity in treatment response. Linear regression (stepwise method) was used to evaluate the factors influencing the effect of the surgery. The regression models' explanatory power was assessed using R^2^ values (range: 0–1), where values closer to 1 indicate stronger relationships between predictor and outcome variables. All significance tests were two‐sided, and the significance level was set at α = 0.05. In figures and tables, symbols denote ^*^ *P* < 0.05, ^*^ *P* < 0.01, ^***^ *P* < 0.001, and ^****^ *P* < 0.0001 unless specified otherwise.

## Author Contributions

Conceptualization: D.A.W.**;** Data curation: G.C., W.Y.F., Q.Z.L., L.Z., K.G.L., Z.L.W., J.L.Z. Formal analysis: Z.Z.**;** Investigation: Z.Z., G.C., W.Y.F., Q.Z.L., L.Z., K.G.L., J.W.D., B.C., X.H., Z.L.W., J.L.Z., J.L.C., Y.H., D.A.W., T.S.**;** Resources: J.L.Z.**;** Validation: G.C., W.Y.F., Q.Z.L., L.Z., K.G.L., J.L.C. Writing – original draft: Z.Z.**;** Writing – review & editing: Z.Z., G.C., W.Y.F., Q.Z.L., L.Z., K.G.L., Y.H., D.A.W., J.C.W., T.S., J.Z.**;** Supervision: Y.H., D.A.W., J.C.W., T.S., J.Z.**;** Project administration: J.L.Z., J.L.C., Y.H., J.C.W., T.S., J.Z.

## Ethics Statement

The studies involving human participants were reviewed and approved by the Ethics Committee of Honghui Hospital, Northern Jiangsu People's Hospital, and Yantaishan Hospital. The participants provided their written informed consent to participate in this study.

## Conflicts of Interest

D.A.W. owns the stock options of the Yantai Dinghao Biotechnology Co., Ltd, and is the unpaid consultant of the Yantai Dinghao Biotechnology Co., Ltd. J.L.Z. and J.L.C. are full‐time employees of Yantai Dinghao Biotechnology Co., Ltd. J.L.Z. and J.L.C. receive salary‐based compensation from Yantai Dinghao Biotechnology Co., Ltd. Non‐corporate authors declare no competing interests. This single‐arm study was funded by Yantai Dinghao Biotechnology Co., Ltd., which also provided the graft material and its related product properties (J.L.Z. and J.L.C.). Data access: All authors had access to all de‐identified raw data across the entire study. Authors who provided raw data assume responsibility for the data they provided. Blinding of MOCART assessments: As this is a single‐arm study, there were no treatment groups to compare, thus eliminating the risk of assessor bias in graft type allocation. The MOCART scoring was performed by three orthopedic surgeons who are not involved in the surgeries, nor are they authors of this manuscript, and were fully independent of both the study team and the sponsor. They had no access to any patient clinical information. They were exposed to follow‐up time information due to inherent time‐dependent post‐operative imaging features, which made it infeasible to maintain complete blinding at the follow‐up time points. Statistical analysis: All primary statistical analyses were conducted by Z.Z., following a prespecified statistical analysis plan finalized. Z.Z. is independent of the funding company and has no financial ties to the sponsor. No company‐affiliated statistician performed or influenced the final analyses. Independent oversight: No independent oversight was involved because it is not required for an investigative study (but only a request for a registration clinical study).

## Supporting information




**Supporting File**: advs77367‐sup‐0001‐SuppMat.docx.

## Data Availability

The data that support the findings of this study are available from the corresponding author upon reasonable request.
