# Supplementary material for: A Clinical Study of Decellularized Tissue Engineering Hyaline Cartilage Graft (dLhCG) for Articular Cartilage Repair
Source: Adv Sci (Weinh). 2026 Oct 13:e23106. Online ahead of print. doi: 10.1002/advs.202523106 (PMC13659700; doi:10.1002/advs.202523106)
Supplement: Supplementary file 1 [file ADVS-9999-e23106-s001.docx]

Supplementary Materials

**•	Multi-Centre Based Investigative Clinical Study of Knee Articular Cartilage Repair via In Situ Implantation of Decellularized Tissue Engineering Hyaline Cartilage Graft - dLhCG**

Zhen Zhang^1†^, Gong Cheng^2†^, Wenyong Fei^3†^, Qiuzhen Liang^4†^, Liang Zhang^4,^*, Kegui Liu^2^, Jihang Dai ^3^, Bo Cong^2^, Xu Hu^1^, Zhonglian Wu^5^, Jialu Zhang^6^, Jinlan Cui^6^, Hang Yao^5,^*, Dong-an Wang^1,7^*, Jingcheng Wang^3,^*, Tao Sun^2,^*, Jiang Zheng^4,^*

*Corresponding author. Email: donganwang@cuhk.edu.hk

**This PDF file includes:**

Methods for preparation of dLhCG

Figure. S1 to S3

Tables S1 to S4

**Methods for preparation of dLhCG**

Healthy porcine chondrocytes were obtained, and a hydrogel culture system was prepared using gelatin, sodium alginate, and other necessary reagents. Gelatin microspheres (diameter 80–120 μm) were first soaked in phosphate-buffered saline (PBS) at 4 °C overnight. They were then mixed uniformly at room temperature with sodium alginate solution and chondrocytes at a ratio of 1 mL sodium alginate solution: 8 ×10⁶ cells: 0.2–0.45 g gelatin microspheres. A custom-designed mold was employed to define the volume of the hydrogel system, and calcium chloride solution was applied to induce crosslinking and solidification. After complete gelation, the mold was removed and the construct was transferred to culture medium for three-dimensional cultivation. At the end of the designated culture period, abundant secretion of type II collagen was observed within the hydrogel.The original hydrogel structure was subsequently disintegrated using sodium citrate solution.

For decellularization, the resulting extracellular matrix secretion system was first rinsed with PBS for 5–20 min and then immersed in 0.5–2% (w/v) Triton X-100 aqueous solution. The treatment was carried out on an orbital shaker at 120–160 rpm at room temperature for 20–50 h. After this step, the samples were rinsed with PBS 3–5 times (4–16 h per rinse) and subjected to alkaline treatment with 0.3–0.4% (w/v) sodium hydroxide solution at 2–8 °C for 20–70 min, during which the vessel was shaken for 15 s every 10 min. Following an additional series of PBS rinses (5–10 times, 4–16 h per rinse), the decellularized material was sealed in sterile containers and sterilized by Cobalt‑60 irradiation at a dose of 25 kGy to eliminate viruses. The final type II collagen‑based extracellular matrix graft was then packaged in double-layer blisters, fulfilling medical‑grade standards.

**Figure S1** Characterization of dLhCG. (A) Representative SEM images showing the surface (i) and cross sectional (ii) morphology of dLhCG.(B) Pore size quantification from SEM images (n=3). (C) Immunohistochemical staining for type I and type II collagen(Scale bar=500 μm). (D, E) Mechanical testing setup for dLhCG.(F) Storage modulus (G’) and loss modulus (G’’) measured by dynamic mechanical analysis at 1 Hz (n=4).

Figure S2.MRI images of all patients before and after dLhCG transplantation at 3-, 6-, and 12-month post-surgery(A-D). The MRI images of six patients after more than 18 months post-surgery (E).Scale bar=2.0cm

**Figure S3**. Preoperative and postoperative blood biomarkers. Abbreviations: Cr, creatinine; Urea, blood urea nitrogen; AST, aspartate aminotransferase; ALT, alanine aminotransferase; WBC, white blood cell count; CRP, C-reactive protein; RBC, red blood cell count; HGB, hemoglobin; PLT, platelet count. (Mixed-effects model,* P<0.05, ** P<0.01)

Table S1.

| **Characteristic** | **Preoperative** | **3 months** | **6 months** | **12 months** | **≥ 18 months** |
| --- | --- | --- | --- | --- | --- |
| **VAS** | 4.35 ± 2.29 | 2.00 ± 1.12 | 1.59 ± 1.33 | 0.53 ± 0.87 | 0.00 ± 0.00 |
| **IKDC** | 52.64 ± 13.76 | 48.87 ± 9.66 | 54.64 ± 12.57 | 61.32 ± 11.33 | 78.35 ± 14.42 |
| **Tegner** | 3.50 ± 1.69 | 4.13 ± 1.64 | 5.63 ± 2.26 | 5.63 ± 2.45 | 5.83 ± 2.23 |
| **Lysholm** | 62.88 ± 19.75 | 70.50 ± 22.51 | 83.63 ± 15.68 | 92.38 ± 11.30 | 88.50 ± 18.28 |
| **MOCART** | - | 55.98 ± 8.36 | 67.84 ± 7.28 | 76.27 ± 10.53 | 80.56 ± 7.35 |

Table S2.

| **Characteristic** | **T4 vs Preoperative** | | **T4 vs T1** | |
| --- | --- | --- | --- | --- |
|  | Mean (95% Cl) | Adjusted P Value | Mean (95% Cl) | Adjusted P Value |
| **VAS** | -4.35 (-1.39 to -7.31) | 0.010 | -2.00 (0.33 to -4.33) | 0.087 |
| **IKDC** | 25.71 (46.74 to 4.68) | 0.023 | 29.49 (49.53 to 9.44) | 0.010 |
| **Tegner** | 2.33 (5.13 to -0.46) | 0.095 | 1.71 (3.41 to 0.006) | 0.049 |
| **Lysholm** | 25.63 (66.54 to -15.29) | 0.225 | 18.00 (50.70 to -14.70) | 0.308 |
| **MOCART** | / | / | 24.58 (39.27 to 9.88) | 0.006 |
| **Characteristic** | **T4 vsT2** | | **T4 vs T3** | |
|  | Mean (95% Cl) | Adjusted P Value | Mean (95% Cl) | Adjusted P Value |
| **VAS** | -1.59 (0.57 to -3.74) | 0.141 | -0.53 (0.25 to -1.31) | 0.181 |
| **IKDC** | 23.71 (43.55 to 3.88) | 0.025 | 17.03 (42.92 to -8.86) | 0.197 |
| **Tegner** | 0.21 (0.84 to -0.42) | 0.689 | 0.21 (1.66 to -1.25) | 0.973 |
| **Lysholm** | 4.88 (17.28 to -7.53) | 0.565 | -3.88 (23.53 to -31.28) | 0.975 |
| **MOCART** | 12.71 (22.32 to 3.11) | 0.017 | 4.28 (14.86 to -6.30) | 0.504 |

Table S3.

| **Item** | **Lysholm** | | | | **Tegner** | | | |
| --- | --- | --- | --- | --- | --- | --- | --- | --- |
|  | **Variables** | **Coefficient** | **P** | **R^2^** | **Variables** | **Coefficient** | **P** | **R^2^** |
| T1 vs. T0 | / | | | | Surgical duration | -0.729 | 0.040 | 0.454 |
| T2 vs. T0 | Age | -0.812 | 0.014 | 0.603 | Age | -0.813 | 0.014 | 0.605 |
| T3 vs. T0 | Gender | 0.737 | 0.037 | 0.468 | Age | -0.750 | 0.032 | 0.490 |
| T3 vs. T1 | / | | | | Disease diagnosis | -0.787 | 0.021 | 0.556 |
| T2 vs. T1 | / | | | | Decfect size | 0.878 | 0.004 | 0.733 |

Table S4.

| **Item** | **IKDC** | | | | **VAS** | | | |
| --- | --- | --- | --- | --- | --- | --- | --- | --- |
|  | **Cluster 1**  **(n=12)** | **Cluster 2**  **(n=5)** | **F** | **P** | **Cluster 1**  **(n=8)** | **Cluster 2**  **(n=9)** | **F** | **P** |
| T1VST0 | 3.85 | -22.07 | 41.306 | <0.001 | -4.88 | -0.11 | 40.314 | <0.001 |
| T2VST0 | 6.62 | -9.09 | 6.570 | 0.022 | -4.63 | -1.11 | 34.459 | <0.001 |
| T3VST0 | 10.59 | 4.10 | 1.316 | 0.269 | -5.63 | -2.22 | 24.994 | <0.001 |
| T3VST2 | 3.97 | 13.19 | 1.894 | 0.189 | -0.75 | -2.11 | 6.400 | 0.023 |
| T3VST1 | 6.75 | 26.16 | 12.674 | 0.003 | -1.00 | -1.11 | 0.042 | 0.841 |
| T2VST1 | 2.77 | 12.98 | 2.291 | 0.151 | 0.25 | -1.00 | 3.365 | 0.087 |
| **Item** | **Lysholm** | | | | **Tegner** | | | |
|  | **Cluster 1**  **(n=4)** | **Cluster 2**  **(n=4)** | **F** | **P** | **Cluster 1**  **(n=3)** | **Cluster 2**  **(n=5)** | **F** | **P** |
| T1VST0 | 29.75 | -14.50 | 17.054 | 0.006 | 1.33 | 0.20 | 4.168 | 0.087 |
| T2VST0 | 36.50 | 5.00 | 12.817 | 0.012 | 4.00 | 1.00 | 16.875 | 0.006 |
| T3VST0 | 49.25 | 9.75 | 39.879 | 0.001 | 4.00 | 1.00 | 10.125 | 0.019 |
| T3VST2 | 19.50 | 24.25 | 0.171 | 0.693 | 0.00 | 0.00 | 0.000 | 1.000 |
| T3VST1 | 12.75 | 4.75 | 1.009 | 0.354 | 2.67 | 0.80 | 5.250 | 0.062 |
| T2VST1 | 6.75 | 19.50 | 1.026 | 0.350 | 2.67 | 0.80 | 5.250 | 0.062 |
| **Item** | **MOCART** | | | | | | | |
|  | **Cluster 1**  **(n=15)** | | **Cluster 2**  **(n=2)** | | **F** | | **P** | |
| T2VST1 | 12.33 | | 8.33 | | 0.653 | | 0.432 | |
| T3VST1 | 22.56 | | 3.33 | | 15.738 | | 0.001 | |
| T3VST2 | 10.22 | | -5.00 | | 12.150 | | 0.003 | |
